# Association of dynamic changes in platelet-to-lymphocyte ratio during chemotherapy with survival in patients with non-small cell lung cancer

**DOI:** 10.3389/fonc.2026.1934583

**Published:** 2026-10-02

**Authors:** Tingshan Xu, Zihang Wu, Jingshan Li, Lingxuan Wang, Baoqiang Weng, Luyang Chen, Xiaohui Chen, Qingyuan Zhu, Peng Liu, Fei He, Yuan Xu

**Affiliations:** 1 Department of Epidemiology and Health Statistics, School of Public Health, Fujian Medical University, Fuzhou, China; 2 Department of Pulmonary and Critical Care Medicine, The Second Affiliated Hospital of Fujian Medical University, Quanzhou, China; 3 NHC Key Laboratory of Etiological Epidemiology of Chronic Diseases with High Incidence in Fujian-Taiwan Area (Co-construction), Fujian Medical University, Fuzhou, China; 4 Fujian Key Laboratory of Lung Stem Cells, The Second Affiliated Hospital of Fujian Medical University, Quanzhou, China

**Keywords:** chemotherapy, dynamic change, non-small cell lung cancer, platelet-to-lymphocyte ratio, prognosis

## Abstract

**Background:**

Inflammatory markers have been reported to be associated with outcomes in non-small cell lung cancer (NSCLC). However, whether early treatment-related changes in these markers provide additional prognostic information remains unclear. This study evaluated the association between dynamic changes in peripheral blood inflammatory markers during chemotherapy and survival outcomes in patients with NSCLC.

**Methods:**

We retrospectively analyzed 479 patients with NSCLC who received chemotherapy. Changes in lymphocyte, platelet, and neutrophil counts, as well as the neutrophil-to-lymphocyte ratio and platelet-to-lymphocyte ratio (PLR), were calculated as absolute differences (D-values) between baseline measurements obtained before the first chemotherapy session and post-chemotherapy measurements obtained within 7 days after the first session. Overall survival (OS) was assessed using Kaplan–Meier analysis and Cox proportional hazards models. Additional Cox models were used to examine the association of PLR D-value with OS after accounting for baseline PLR and clinical covariates. A prognostic nomogram was constructed as an exploratory risk-estimation model.

**Results:**

Greater changes in the evaluated hematological markers were associated with shorter OS in Kaplan–Meier analysis. In the primary multivariable Cox regression, high PLR D-value was associated with shorter OS (hazard ratio, 1.422; 95% confidence interval, 1.001–2.020; *P* = 0.049), together with sex, anemia status, and tumor extent. After adjustment for baseline PLR and clinical covariates, however, PLR D-value was no longer significantly associated with OS when analyzed either as a continuous variable (HR per 50-unit increase, 1.010; 95% CI, 0.947–1.078; *P* = 0.762) or using the original high/low classification (HR, 1.31; 95% CI, 0.91–1.89; *P* = 0.145). The nomogram had an apparent C-index of 0.630 and an optimism-corrected C-index of 0.618 after 1,000 bootstrap resamples.

**Conclusions:**

Greater changes in PLR during chemotherapy were associated with shorter OS in the primary categorical analysis. However, this association was attenuated after baseline PLR and clinical factors were taken into account, indicating that the additional prognostic contribution of PLR D-value may be limited. The nomogram should be considered exploratory and requires external validation before clinical application.

## Background

Lung cancer (LC) remains one of the most commonly diagnosed cancers and the leading cause of cancer-related mortality in China. According to national cancer statistics in China, approximately 1.06 million new lung cancer cases were diagnosed in 2022. Lung cancer caused nearly 0.73 million deaths, accounting for 28.5% of all cancer-related deaths (1). Non-small cell lung cancer (NSCLC) is the most prevalent form of LC, with adenocarcinoma and squamous cell carcinoma being the dominant histological subtypes (2). Although surgery, chemotherapy, radiotherapy, and immunotherapy have considerably improved the survival of patients with NSCLC, the overall prognosis remains suboptimal (3). Chemotherapy remains an important component of NSCLC treatment, either alone or as part of multimodal therapy. However, due to pronounced inter-patient heterogeneity and intrinsic tumor biological variability, responses to chemotherapy and clinical outcomes vary markedly among individuals. Consequently, there is an urgent need to identify reliable biomarkers that can support risk stratification and clinical assessment in patients with NSCLC.

Although molecular and tumor-related biomarkers have been increasingly investigated for prognostic assessment and treatment selection in NSCLC (4), their clinical application often requires specialized assays and additional resources. In contrast, inflammatory markers including platelet, lymphocyte, and neutrophil counts are routinely available, cost-effective, and reproducible in clinical practice. Indices such as the neutrophil-to-lymphocyte ratio (NLR) and the platelet-to-lymphocyte ratio (PLR) provide readily accessible measures of systemic immune-inflammatory status. Beyond their role in hemostasis, platelets participate in tumor progression and tumor–immune interactions. They can shield circulating tumor cells from immune recognition and modulate antitumor immunity through interactions with immune cells, including natural killer cells and T cells. Platelet-derived mediators may further suppress antitumor immune activity (5, 6), whereas lymphocytes are central components of host immune surveillance (7, 8). Thus, PLR may provide an indirect measure of the balance between platelet-associated protumor processes and lymphocyte-mediated antitumor immunity. Accumulating evidence has demonstrated that elevated NLR and PLR levels are associated with poor prognosis across several malignancies, including NSCLC (9–12).

Most previous studies have focused on the association between single static measurements, taken either before or after treatment, and patient prognosis. However, chemotherapy can induce changes in hematopoietic and immune responses during treatment (13, 14). Therefore, static assessments may not fully characterize treatment-related changes in systemic immune-inflammatory status. Changes in circulating blood-cell counts and their derived indices during chemotherapy may provide information that is not captured by a single measurement and may also be associated with clinical outcomes (15).

Therefore, this study aimed to investigate whether changes in peripheral blood immune-inflammatory markers during chemotherapy were associated with overall survival (OS) in patients with NSCLC. We focused particularly on PLR D-value because PLR integrates changes in platelet and lymphocyte counts, two components with distinct roles in tumor biology and host immunity. The magnitude of change was quantified using the absolute difference between baseline and post-chemotherapy measurements.

## Methods

### Study cohort

This retrospective study analyzed clinical data from 479 patients with pathologically confirmed NSCLC who underwent chemotherapy at the First Affiliated Hospital of Fujian Medical University and the Second Affiliated Hospital of Fujian Medical University between January 2013 and April 2025. Patients were enrolled based on the following inclusion criteria (1): Pathologically confirmed primary NSCLC (2); Receipt of chemotherapy (3); Absence of other malignancies or life-threatening diseases (4); No contraindications to chemotherapy or postoperative adjuvant chemotherapy (5); No treatment with corticosteroids or immunosuppressants (which may affect peripheral blood cell counts) during the peri-chemotherapy period (6); Availability of the baseline and post-chemotherapy hematological measurements required for calculation of the D-values, together with survival follow-up information. Patients who received immunotherapy, molecularly targeted therapy, or other systemic anticancer treatments between the baseline and post-chemotherapy hematological assessments were excluded to minimize treatment-related confounding.

Additionally, patients with the following complications were excluded to avoid interference with blood counts or survival assessment: pulmonary embolism, confirmed pulmonary infection, acute myocardial infarction, severe hepatic or renal dysfunction, and cerebrovascular accidents.

This study was approved by the Ethics Committee of the Fujian Medical University (No. 2026-171) and the Second Affiliated Hospital of Fujian Medical University (No. 2025-062).

### Data collection

Clinical data were retrospectively extracted from the hospital electronic medical record system, including patient demographics (sex, age, body mass index [BMI]), anemia status, fasting blood glucose, histological type, clinical stage, tumor location, and maximum tumor diameter. Clinical stage, when available, was determined according to the 8th edition of the Tumor-Node-Metastasis (TNM) Classification of Malignant Tumors (Union for International Cancer Control/American Joint Committee on Cancer, UICC/AJCC, 2017), integrating imaging and pathological findings.

Peripheral blood immune-inflammatory markers, including LYM, PLA, and NEU counts, were measured at baseline before the first chemotherapy session and again within 7 days after the first chemotherapy session. NLR and PLR were calculated separately at each time point from the corresponding blood cell counts. The absolute difference (D-value), representing the magnitude of change irrespective of direction, was defined as:


D-value=|Baseline value−Post-chemotherapy value|

### Follow-up

Patients were followed through telephone interviews and outpatient clinical records at approximately six-month intervals. The recorded information included survival status, disease progression, and available treatment information. The primary endpoint was overall survival (OS), defined as the time from the first chemotherapy session to death or the last follow-up. For surviving patients, the follow-up cutoff date was April 30, 2025. For patients lost to follow-up, the cutoff date was based on the last successful telephone contact or the date of the last hospital discharge recorded in the medical records.

### Statistical analysis

All statistical analysis were performed using SPSS (version 25.0) and R software (version 4.3.1). Patients with missing data for a specific variable were excluded from analysis involving that variable. In descriptive analysis, missing data were reported as an “unknown” category when applicable.

The optimal cutoff values for NLR, PLR, LYM, PLA, and NEU D-values were determined using X-tile software (version 3.6.1) based on the minimum P-value method. Patients were categorized into High D-value and Low D-value groups. Because these cutoffs were derived and evaluated in the same study population, the corresponding categorical analysis were considered exploratory. Categorical clinical characteristics were compared between groups using the chi-square (χ²) test or Fisher’s exact test. Survival curves were generated using the Kaplan-Meier method and compared using the log-rank test.

To identify prognostic factors, variables with P < 0.05 in univariable Cox regression analysis, along with core indicators (NLR D-value and PLR D-value), were considered for inclusion in a multivariable Cox proportional hazards model. To minimize redundant information between closely related measures of disease extent, overall clinical stage was retained rather than distant metastasis. To reduce redundancy among the hematological D-values, NLR D-value and PLR D-value were retained as representative composite indices. Hazard ratios (HRs) and 95% confidence intervals (CIs) were calculated.

To further assess whether PLR D-value provided prognostic information beyond baseline PLR, nested Cox regression models were constructed in the complete-case population by sequentially adding baseline PLR and PLR D-value to a clinical model comprising sex, clinical stage, tumor location, tumor size, histological type, anemia status, and chemotherapy regimen. The same complete-case population and the same set of clinical covariates were used across all nested models to ensure direct comparability. Baseline PLR and PLR D-value were analyzed as continuous variables, both expressed per 50-unit increase, and incremental model fit was evaluated using likelihood-ratio tests. A sensitivity analysis was additionally performed using the original high/low PLR D-value classification. Because the absolute PLR D-value does not preserve the direction of change, sensitivity analysis were also performed using signed PLR change (post-chemotherapy PLR minus baseline PLR) and by comparing patients with increased versus decreased PLR. Potential heterogeneity according to disease stage was further examined in the complete-case population by grouping patients as early stage (I–II) or advanced stage (III–IV) and testing the interaction between stage group and continuous PLR D-value (per 50-unit increase) in the Cox model.

A nomogram was constructed based on variables that remained statistically significant in the primary multivariable Cox model to predict 1-, 3-, and 5-year survival probabilities. The model’s performance was evaluated using the concordance index (C-index) for discrimination, time-dependent receiver operating characteristic (ROC) curves, and calibration curves to assess agreement between predicted and observed survival probabilities. The proportional hazards assumption was assessed using Schoenfeld residuals. When evidence of non-proportional hazards was identified, stratified Cox models were additionally used as sensitivity analysis. Internal validation of the nomogram was performed using bootstrap resampling with 1,000 repetitions to obtain the optimism-corrected C-index and calibration curves. Because the nomogram underwent internal validation only and was not externally validated, its predictive performance was interpreted as exploratory. These analyses were primarily implemented using the rms, survival, and timeROC packages in R. All statistical tests were two-sided, and *P* < 0.05 was considered statistically significant.

## Results

### Cohort study

A total of 479 patients with NSCLC were enrolled in this study. The clinical and demographic characteristics are summarized in **Table 1**. The mean age was 64.25 ± 9.04 years, including 371 males (77.5%) and 108 females (22.5%). Adenocarcinoma was the predominant histological type (57.8%). Clinical stage III–IV disease was recorded in 55.8% of the total cohort, although clinical stage was unavailable for 142 patients (29.6%). Tumor involvement was confined to a single lobe in 62.8% of patients. The mean maximum tumor diameter was 3.45 ± 2.58 cm. The majority of patients (58.5%) received combination chemotherapy.

**Table 1:** Baseline characteristics of patients with NSCLC (N = 479).

Factors		n/mean ± SD/median (IQR)	Percentage (%)
Sex			
Male		371	77.5
Female		108	22.5
Age (years)		64.25 ± 9.04	
<65		239	49.9
≥65		240	50.1
Clinical stage			
I		23	4.8
II		47	9.8
III		124	25.9
IV		143	29.9
Unknown		142	29.6
Distant metastasis			
Yes		144	30.1
No		335	69.9
Histological type			
Adenocarcinoma		277	57.8
Squamous cell carcinoma		178	37.2
Unknown		24	5.0
Tumor location			
Single lobe		301	62.8
Upper lobe		182	38.0
Middle lobe		19	4.0
Lower lobe		100	20.8
Bilobar involvement		145	30.3
Whole lung		19	4.0
Unknown		14	2.9
Tumor maximum size (cm)		3.45 ± 2.58	
<4.16		174	36.3
4.16-5.20		34	7.1
≥5.20		68	14.2
Unknown		203	42.4
BMI		22.55 ± 2.96	
<18.5		20	4.2
18.5-23.9		212	44.3
24.0-27.9		80	16.7
≥28		20	4.2
Unknown		147	30.7
Hemoglobin (g/L)		128.91 ± 16.67	
Anemia status			
No		374	78.3
Yes		105	21.7
Fasting blood glucose		5.62 ± 1.73	
Normal		324	67.6
Abnormal		116	24.2
Unknown		39	8.1
Chemotherapy regimen			
Combination therapy		280	58.5
Platinum + antimetabolite		89	18.6
Platinum + plant-based		180	37.6
Other		11	2.3
Monotherapy		199	41.5
Platinum		167	34.9
Antimetabolite		23	4.8
Plant-based		9	1.9
		Median (IQR)	
NLR D-value		1.70 (0.70–3.75)	
PLR D-value		60.98 (26.76–122.87)	
LYM D-value (10^9^/L)		0.40 (0.17–0.70)	
PLA D-value (10^9^/L)		61.00 (27.00–107.00)	
NEU D-value (10^9^/L)		1.99 (0.90–3.80)	

According to the clinical anemia diagnostic standards in China, anemia is defined as a Hb level of less than 120 g/L for adult males and less than 110 g/L for adult females. Fasting blood glucose levels prior to treatment are categorized based on the WHO diabetes diagnostic standards: normal (3.9-6.1 mmol/L) and abnormal (<3.9 or >6.1 mmol/L). All percentages are based on the total cohort (N = 479). D-value is the absolute difference between the baseline value and the post-chemotherapy value. NLR, neutrophil-to-lymphocyte ratio; PLR, platelet-to-lymphocyte ratio; LYM, lymphocyte count; PLA, platelet count; NEU, neutrophil count.

As all hematological D-values were skewed, they were reported as medians with interquartile ranges (IQRs): NLR D-value: 1.70 (0.70–3.75); PLR D-value, 60.98 (26.76–122.87); LYM D-value, 0.40 (0.17–0.70); PLA D-value, 61.00 (27.00–107.00); and NEU D-value, 1.99 (0.90–3.80). The median OS of the cohort was 22.01 months (IQR, 10.55–40.74 months). All patients included in the study survived beyond 7 days after the first chemotherapy session.

### Determination of cutoff values for NLR, PLR, LYM, PLA, and NEU D-values

The optimal cutoff values for the hematological D-values were determined using X-tile software based on data from the 479 patients. The optimal cutoff values were 0.6 for LYM D-value, 28.0 for PLA D-value, 1.6 for NEU D-value, 1.5 for NLR D-value and 45.2 for PLR D-value (**Figure 1**). Based on these thresholds, patients were classified into low- and high-D-value groups as follows: NLR D-value: Low (<1.5) and High (≥1.5); PLR D-value: Low (<45.2) and High (≥45.2); LYM D-value: Low (<0.6) and High (≥0.6); PLA D-value: Low (<28.0) and High (≥28.0); NEU D-value: Low (<1.6) and High (≥1.6).

**Figure 1:**
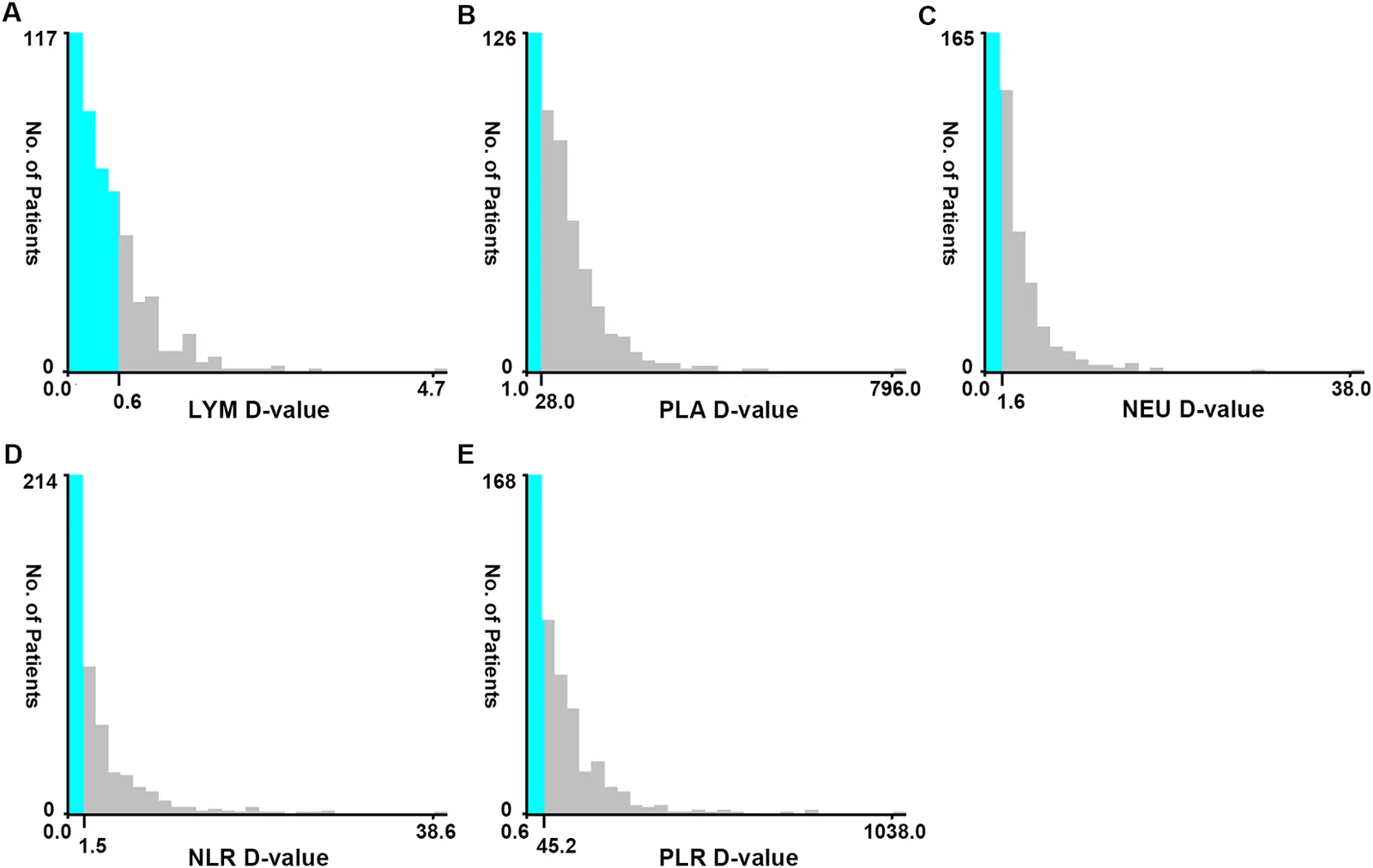
X-tile determination of optimal cutoff values for hematological D-values. Histograms illustrate the distributions of hematological D-values in patients with non-small cell lung cancer (NSCLC; n = 479). The optimal cutoff values were determined using X-tile analysis. **(A)** Lymphocyte (LYM) D-value, cutoff = 0.6; **(B)** platelet (PLA) D-value, cutoff = 28.0; **(C)** neutrophil (NEU) D-value, cutoff = 1.6; **(D)** neutrophil-to-lymphocyte ratio (NLR) D-value, cutoff = 1.5; and **(E)** platelet-to-lymphocyte ratio (PLR) D-value, cutoff = 45.2.

### Associations between hematological D-values and clinical characteristics

The chi-square test results (**Table 2**) revealed significant associations between hematological D-values and certain clinicopathological features. Except for PLR D-value, the other four indicators (NLR, PLA, LYM, and NEU D-values) differed significantly by sex (*P* < 0.05). The maximum tumor diameter was significantly associated with all D-values except for PLA D-value (*P* < 0.05). PLA D-value was associated with clinical stage (*P* = 0.029) and histological type (*P* < 0.001), while NLR D-value was also associated with histological type (*P* = 0.016). Regarding treatment, combination chemotherapy was associated with LYM D-value (*P* = 0.009), whereas the combination chemotherapy regimen was associated with PLR D-value (*P* = 0.005). No other significant associations were observed between the evaluated clinical characteristics and hematological D-values.

**Table 2:** Relationship between hematological D-values and baseline clinical characteristics.

Factors	NLR D-value			PLR D-value			LYM D-value			PLA D-value			NEU D-value		
	Low	High	*P*	Low	High	*P*	Low	High	*P*	Low	High	*P*	Low	High	*P*
Sex															
Male	159	212	0.008	146	225	0.102	235	136	0.015	79	292	<0.001	147	224	0.006
Female	62	46		52	56		82	26		41	67		59	49	
Age (years)															
<65	106	133	0.434	101	138	0.682	150	89	0.115	63	176	0.510	103	136	0.968
≥65	115	125		97	143		167	73		57	183		103	137	
Clinical stage															
I	14	9	0.346	12	11	0.199	17	6	0.425	9	14	0.029	11	12	0.339
II	25	22		23	24		32	15		16	31		22	25	
III	57	67		47	77		73	51		28	96		53	71	
IV	62	81		50	93		93	50		25	118		50	93	
Distant metastasis															
No	163	172	0.092	147	188	0.085	226	109	0.365	90	245	0.162	144	191	0.989
Yes	58	86		51	93		91	53		30	114		62	82	
Histological type															
Adenocarcinoma	144	133	0.016	124	153	0.133	190	87	0.136	87	190	<0.001	126	151	0.290
Squamous cell carcinoma	72	106		67	111		110	68		29	149		72	106	
Tumor location															
Single lobe	132	169	0.433	127	174	0.993	199	102	0.792	77	224	0.924^*^	130	171	0.882
Bilobar involvement	73	72		62	83		96	49		35	110		59	86	
Whole lung	9	10		8	11		14	5		5	14		8	11	
Maximum tumor diameter (cm)															
<4.16	83	91	0.020	77	97	0.018	120	54	0.004	42	132	0.397	81	93	0.040
4.16-5.20	14	20		8	26		14	20		8	26		12	22	
≥5.20	19	49		20	48		38	30		11	57		20	48	
BMI															
<18.5	9	11	0.984	6	14	0.101	16	4	0.463	6	14	0.962	8	12	0.903
18.5-23.9	100	112		91	121		145	67		56	156		99	113	
24.0-27.9	39	41		40	40		61	19		23	57		39	41	
≥28	10	10		13	7		14	6		5	15		10	10	
Anemia status															
No	181	193	0.061	163	211	0.059	245	129	0.558	100	274	0.108	167	207	0.170
Yes	40	65		35	70		72	33		20	85		39	66	
Fasting blood glucose															
Normal	152	172	0.304	134	190	0.629	215	109	0.740	82	242	0.318	135	189	0.914
Abnormal	48	68		45	71		75	41		24	92		49	67	
Chemotherapy regimen															
Monotherapy	100	99	0.128	87	112	0.372	145	54	0.009	57	142	0.126	92	107	0.229
Combination	121	159		111	169		172	108		63	217		114	166	
Combination regimen															
Platinum + antimetabolite	40	49	0.609	25	64	0.005	48	41	0.116	18	71	0.499	31	58	0.280
Platinum + plant-based	75	105		83	97		115	65		43	137		75	105	

*P*-values were calculated using Pearson’s chi-square test or *Fisher’s exact test (where expected cell counts were < 5). Patients with missing clinical data were excluded from the respective analysis. *P* < 0.05 was considered statistically significant. NLR, neutrophil-to-lymphocyte ratio; PLR, platelet-to-lymphocyte ratio; LYM, lymphocyte count; PLA, platelet count; NEU, neutrophil count; D-value, absolute difference between the baseline value and the post-chemotherapy value.

### Association between hematological D-values and OS in NSCLC patients

Kaplan-Meier survival analysis demonstrated significant differences in OS between the high- and low-D-value groups for all evaluated hematological markers. Patients in the high-D-value groups for NLR, PLR, LYM, PLA, and NEU had shorter OS than those in the corresponding low-D-value groups (**Figure 2**).

**Figure 2:**
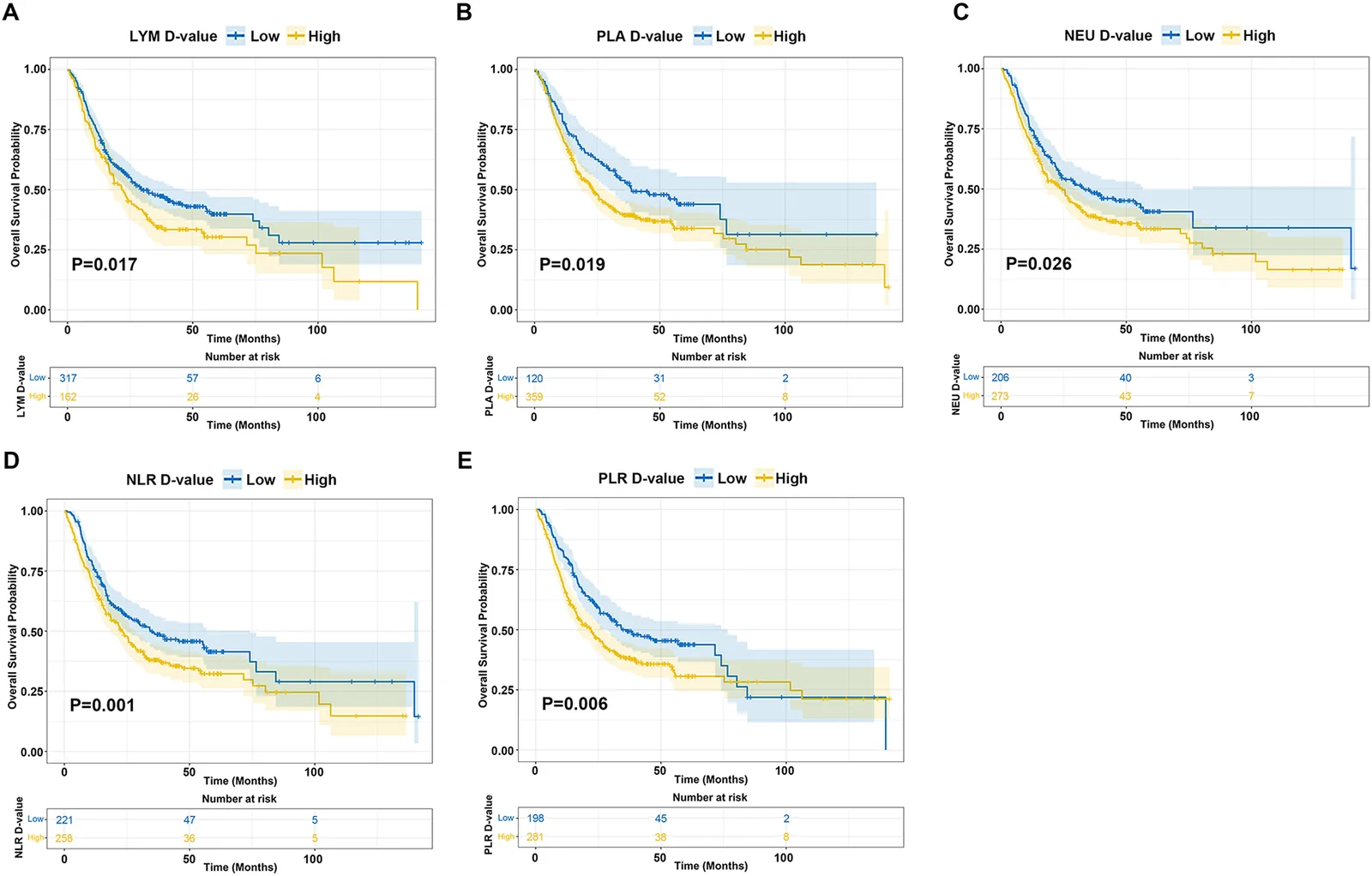
Kaplan–Meier survival curves for overall survival (OS) stratified by hematological D-values. Overall survival was compared between high and low D-value groups using Kaplan–Meier analysis and the log-rank test. **(A)** LYM D-value; **(B)** PLA D-value; **(C)** NEU D-value; **(D)** NLR D-value; and **(E)** PLR D-value.

Among primary hematological indicators, the low NEU D-value group showed a survival advantage, with a median overall survival (mOS) of 33.08 months (95% CI: 14.52–51.64), longer than the 24.74 months observed in the high NEU D-value group (95% CI: 19.66–29.81; *P* = 0.026). Similarly, patients in the low LYM D-value group had a superior survival (mOS: 29.96 months, 95% CI: 20.36–39.56) compared to the high LYM D-value group (mOS: 21.62 months, 95% CI: 16.37–26.87; *P* = 0.017).The low PLA D-value was also associated with a significant survival advantage (mOS: 38.63 months, 95% CI: 19.36–57.90) over the high PLA D-value group (mOS: 23.10 months, 95% CI: 18.74–27.45; *P* = 0.019).

Similar differences were observed for the composite immune-inflammatory indices. The high NLR D-value group had a shorter mOS (23.03 months, 95% CI: 18.14–27.92) than the low NLR D-value group (34.26 months, 95% CI: 15.80–52.73; *P* = 0.010). Likewise, the low PLR D-value group achieved a better prognosis, with an mOS of 36.10 months (95% CI: 18.71–53.50), compared to the high PLR D-value group (mOS: 22.01months, 95% CI: 16.27–27.75; *P* = 0.006).

### Univariable analysis of prognostic factors for OS

Univariable Cox regression analysis identified several clinical factors and dynamic hematological indicators that were significantly associated with OS (**Table 3**). Female patients had a significantly lower risk of death than males (HR = 0.501, 95% CI: 0.365–0.687; P < 0.001). In contrast, advanced clinical stage (overall P < 0.001), presence of distant metastasis (HR = 2.483; P < 0.001), squamous cell carcinoma histology (HR = 1.364; P = 0.012), and larger tumor diameter (overall P < 0.001) were associated with poorer OS. Patients with anemia also exhibited a significantly increased risk of death (HR = 2.005; P < 0.001). All five hematological D-values were associated with OS. High NLR D-value (HR = 1.365; P = 0.010), High PLR D-value (HR = 1.399; P = 0.006), and High LYM, PLA, and NEU D-values (all P < 0.05) were associated with shorter OS. Combination chemotherapy was also associated with OS in the univariable analysis (HR = 1.395, 95% CI: 1.091–1.784; P = 0.008), whereas age, BMI, and fasting blood glucose were not significantly associated with OS.

**Table 3:** Cox regression analysis of factors associated with OS in patients with NSCLC.

Factors	Univariable analysis		Multivariable analysis	
	HR (95%CI)	*P*	HR (95%CI)	*P*
Sex				
Male	1 (ref)	–	1 (ref)	–
Female	0.501(0.365, 0.687)	<0.001	0.511(0.315, 0.831)	0.007
Age				
<65	1 (ref)	–	–	–
≥65	1.231(0.973,1.557)	0.083	–	–
Clinical stages				
I	1 (ref)	–	1 (ref)	–
II	2.904(0.846, 9.969)	0.090	1.327(0.279, 6.309)	0.722
III	7.613(2.405, 24.099)	0.001	3.081(0.735, 12.918)	0.124
IV	11.058(3.512, 34.819)	<0.001	4.074(0.989, 16.786)	0.052
Distant metastasis				
No	1 (ref)	–	–	–
Yes	2.483(1.959, 3.147)	<0.001	–	–
Histological type				
Adenocarcinoma	1 (ref)	–	1 (ref)	–
Squamous cell carcinoma	1.363(1.071, 1.736)	0.012	0.813 (0.557–1.188)	0.285
Other	1.297(0.321, 5.238)	0.715		
Tumor location				
Single lobe	1 (ref)	–	1 (ref)	–
Bilobar involvement	1.320(1.025, 1.700)	0.031	1.080(0.737, 1.584)	0.693
Whole lung	1.591(0.922, 2.748)	0.095	2.213(1.099, 4.458)	0.026
Maximum tumor diameter				
<4.16	1 (ref)	–	1 (ref)	–
4.16-5.20	1.808(1.202, 2.721)	0.004	1.433(0.865, 2.374)	0.162
≥5.20	1.894(1.360, 2.638)	<0.001	1.452(0.978, 2.155)	0.064
BMI				
<18.5	1 (ref)	–	–	–
18.5-23.9	0.646(0.355, 1.178)	0.154	–	–
24.0-27.9	0.528(0.273, 1.025)	0.059	–	–
≥28	0.396(0.156, 1.008)	0.052	–	–
Anemia status				
No	1 (ref)	–	1 (ref)	**-**
Yes	2.005(1.544, 2.603)	<0.001	1.780(1.207, 2.625)	0.004
Fasting blood-glucose				
Normal	1 (ref)	–	–	–
Abnormal	1.022(0.777, 1.343)	0.879	–	–
Chemotherapy regimen				
Monotherapy	1 (ref)	–	1 (ref)	–
Combination therapy	1.395(1.091, 1.784)	0.008	0.757(0.520, 1.102)	0.147
NLR D-value				
Low	1 (ref)	–	1 (ref)	–
High	1.365(1.078, 1.730)	0.010	1.042(0.739, 1.468)	0.816
PLR D-value				
Low	1 (ref)	–	1 (ref)	–
High	1.399(1.100, 1.780)	0.006	1.422(1.001, 2.020)	0.049
LYM D-value				
Low	1 (ref)	–	–	–
High	1.338(1.054,1.699)	0.017	–	–
PLA D-value				
Low	1 (ref)	–	–	–
High	1.399(1.055, 1.855)	0.020	–	–
NEU D-value				
Low	1 (ref)	–	–	–
High	1.312(1.032, 1.667)	0.027	–	–

Variables with *P* < 0.05 in univariable Cox regression analysis, together with NLR D-value and PLR D-value, were considered for inclusion in the multivariable Cox proportional hazards model. Clinical stage was retained rather than distant metastasis to reduce redundancy in measures of disease extent, and NLR D-value and PLR D-value were retained as representative composite hematological indices.HR, hazard ratio; CI, confidence interval; ref, reference category; NLR, neutrophil-to-lymphocyte ratio; PLR, platelet-to-lymphocyte ratio; LYM, lymphocyte count; PLA, platelet count; NEU, neutrophil count;.

### Multivariable Cox regression analysis

A multivariable Cox proportional hazards model was constructed to evaluate factors associated with OS after multivariable adjustment (**Table 3**). Clinical variables with *P* < 0.05 in the univariable analysis were considered for inclusion, with clinical stage retained rather than distant metastasis to reduce redundancy in measures of disease extent. NLR D-value and PLR D-value were retained as representative composite hematological indices. The analysis showed that female patients had a significantly lower risk of death than males (HR = 0.511, 95% CI: 0.315–0.831, *P* = 0.007). Anemia remained significantly associated with a higher risk of death (HR = 1.780, 95% CI: 1.207–2.625, *P* = 0.004). Patients with whole-lung involvement had significantly worse outcomes than those with single-lobe involvement (HR = 2.213, 95% CI: 1.099–4.458, *P* = 0.026). High PLR D-value was also associated with an increased risk of death compared with Low PLR D-value (HR = 1.422, 95% CI: 1.001–2.020, *P* = 0.049).

Because baseline PLR may itself carry prognostic information, we further examined whether PLR D-value provided additional prognostic information after accounting for baseline PLR. All nested models were fitted in the same complete-case population of 214 patients, including 156 deaths, using the same set of clinical covariates (**Supplementary Table S1**). Adding baseline PLR to the clinical model improved model fit (likelihood-ratio test, *P* = 0.032), and higher baseline PLR was associated with poorer OS (HR per 50-unit increase = 1.084, 95% CI: 1.010–1.164, *P* = 0.026). After PLR D-value was further added to the model, however, its association with OS was not statistically significant (HR per 50-unit increase = 1.010, 95% CI: 0.947–1.078, *P* = 0.762), and model fit was not further improved (likelihood-ratio test, *P* = 0.764). When the original High/Low PLR D-value classification was used, High PLR D-value was likewise not significantly associated with OS after adjustment for baseline PLR and the same clinical covariates (HR = 1.31, 95% CI: 0.91–1.89, *P* = 0.145; **Supplementary Table S2**).

Direction-sensitive analyses further showed that a greater positive signed PLR change was associated with poorer OS (HR per 50-unit increase = 1.04, 95% CI: 1.00–1.09, *P* = 0.044), whereas the comparison between patients with increased and decreased PLR was not statistically significant (HR = 1.33, 95% CI: 0.94–1.88, *P* = 0.110). After stratification by chemotherapy regimen, the association for signed PLR change was attenuated and was of borderline statistical significance (*P* = 0.051). No significant interaction was observed between stage group and continuous PLR D-value (*P* for interaction = 0.930), providing no evidence that the association between PLR D-value and OS differed between early- and advanced-stage disease. This conclusion was unchanged in the sensitivity analysis accounting for the non-proportional hazards of chemotherapy regimen.

### Exploratory prognostic model performance

Based on variables that remained statistically significant in the multivariable Cox model, a visual nomogram incorporating sex, tumor location, anemia status, and PLR D-value was constructed to estimate 1-, 3-, and 5-year OS probabilities. The nomogram predicted 1-, 3-, and 5-year survival probabilities for patients with NSCLC (**Figure 3**). The apparent C-index of the nomogram was 0.630. After 1,000 bootstrap resamples, the optimism-corrected C-index was 0.618. Time-dependent ROC analysis showed that the AUC values for 1-, 3-, and 5-year OS were 0.656 (95% CI: 0.567–0.746), 0.727 (95% CI: 0.630–0.829), and 0.783 (95% CI: 0.660–0.906) (**Figure 4**). The calibration curves showed agreement between predicted and observed survival probabilities at 1, 3, and 5 years (**Figure 5**).

**Figure 3:**
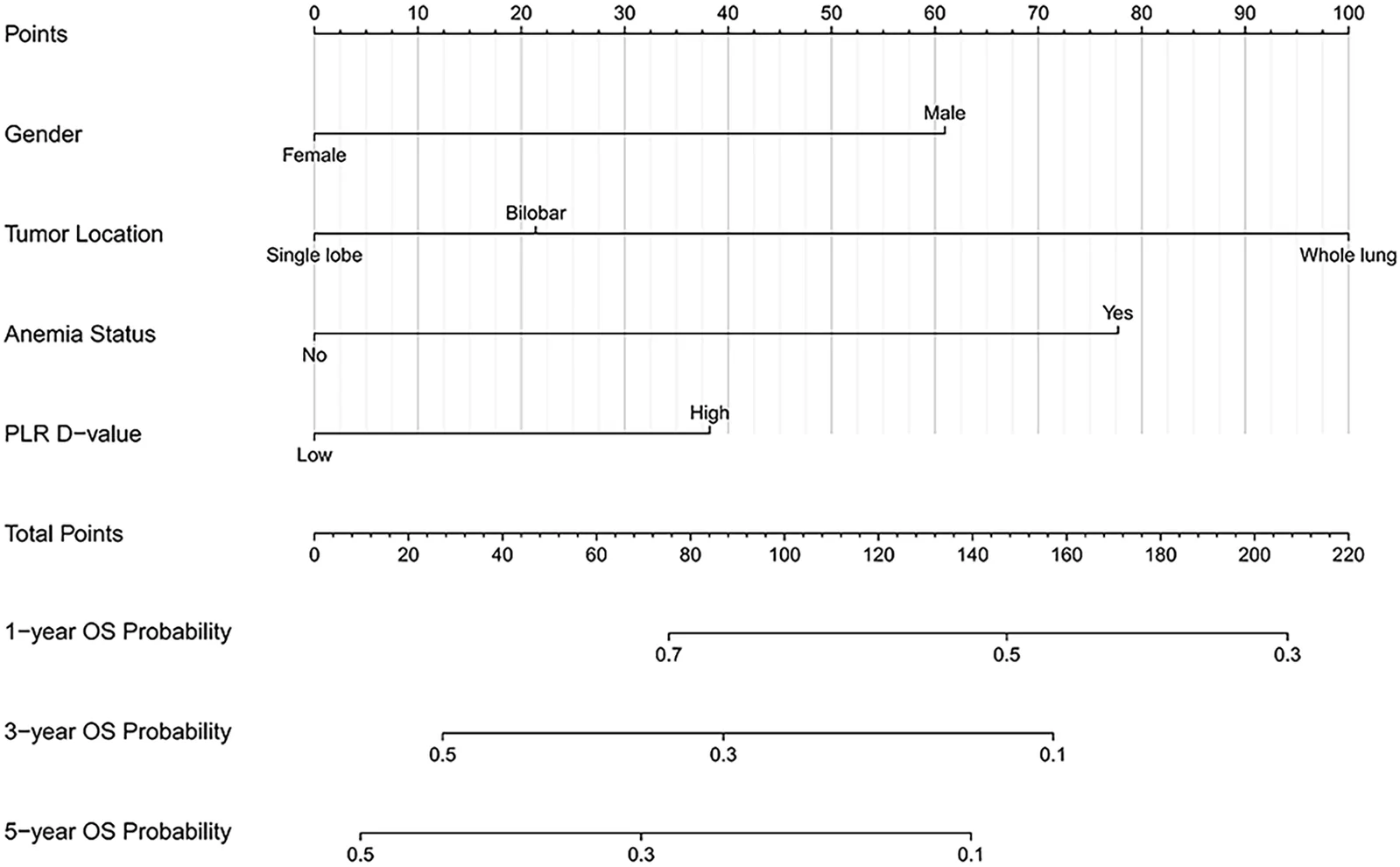
Exploratory nomogram for estimating overall survival in patients with NSCLC. The nomogram incorporated sex, tumor location, anemia status, and PLR D-value to estimate 1-, 3-, and 5-year overall survival probabilities.

**Figure 4:**
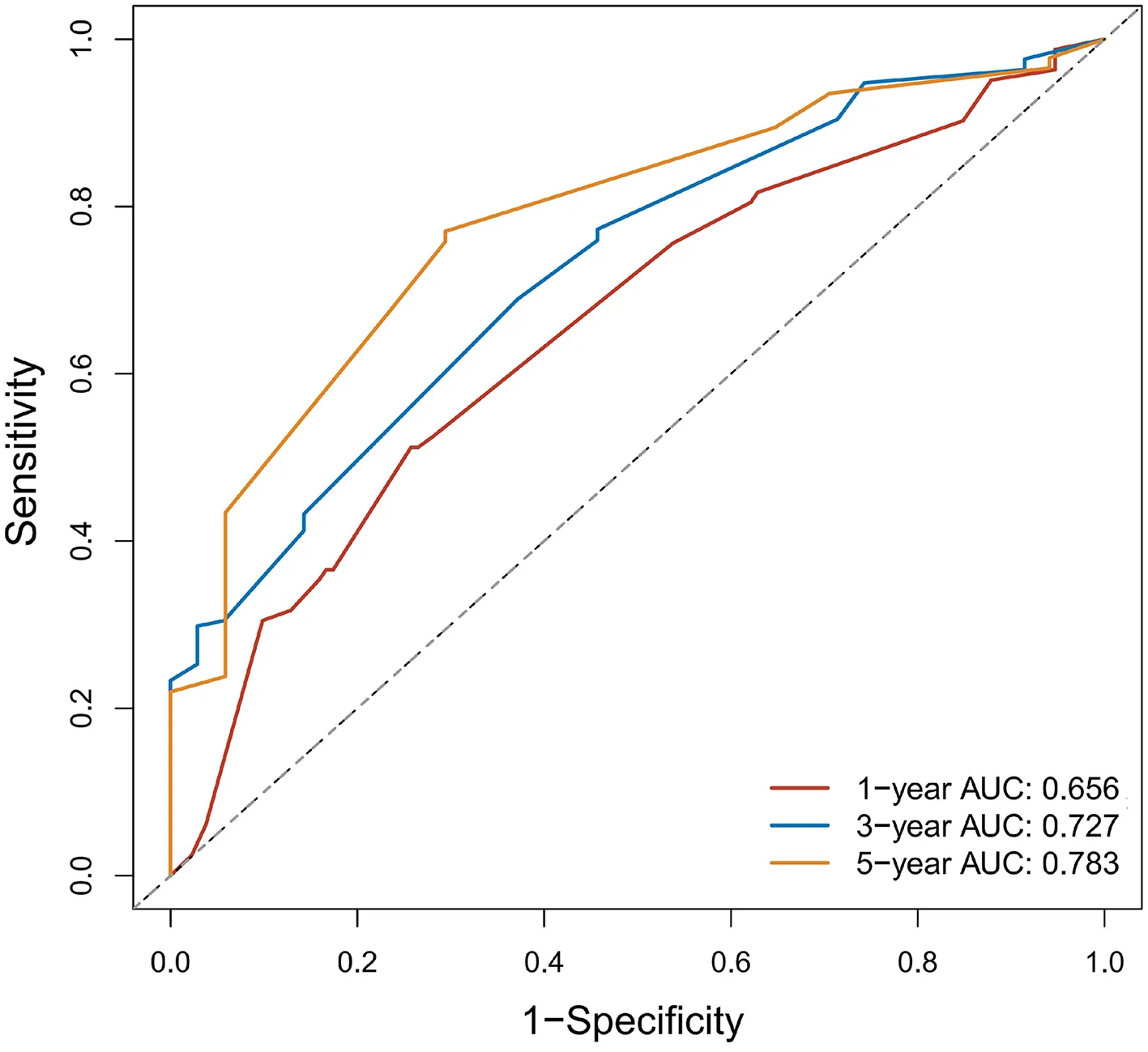
Time-dependent receiver operating characteristic curves of the prognostic nomogram. Time-dependent ROC curves were used to assess the discriminative performance of the nomogram for 1-, 3-, and 5-year overall survival.

**Figure 5:**
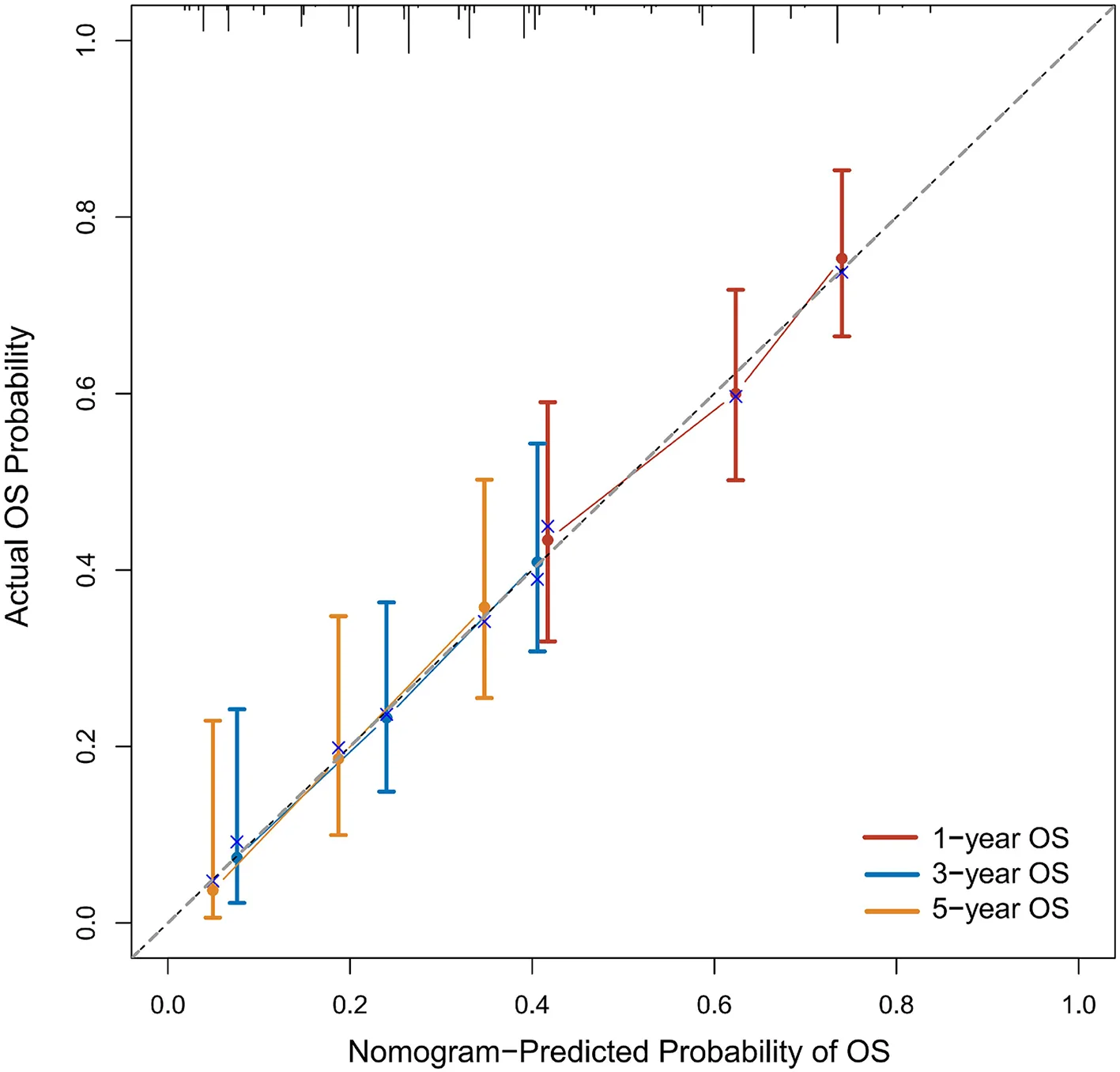
Calibration curves for the exploratory prognostic nomogram. Calibration curves were generated using 1,000 bootstrap resamples to compare predicted and observed survival probabilities at 1, 3, and 5 years.

## Discussion

This study evaluated the association between dynamic changes in peripheral blood inflammatory markers during chemotherapy and survival outcomes in patients with NSCLC. We found that greater fluctuations in several hematological markers were associated with shorter OS. In the primary multivariable analysis, high PLR D-value was associated with an increased risk of death, together with sex, anemia status, and tumor involvement. However, this association was attenuated after further adjustment for baseline PLR and clinical factors. Female patients exhibited better outcomes, whereas patients with anemia or whole-lung tumor involvement had poorer survival. These findings indicate that the prognostic relevance of PLR changes should be interpreted in the context of baseline PLR and established clinical factors.

Previous studies on immune-inflammatory markers have largely focused on static indicators measured at a single time point in NSCLC (16–19). More recent studies have also examined dynamic changes in peripheral blood indices during treatment (15). However, single-time-point assessments may not fully characterize treatment-related changes in systemic immune-inflammatory status. In this study, we quantified the magnitude of these changes using the absolute difference (D-value) of immune-inflammatory markers. Higher D-values were associated with poorer OS, suggesting that larger hematological changes during chemotherapy may be associated with less favorable clinical outcomes. Furthermore, composite indices such as NLR and PLR incorporate information from more than one circulating blood-cell component and have been widely investigated as prognostic markers in NSCLC (16, 17, 19).

Chemotherapy can alter hematopoiesis and systemic immune-inflammatory responses during treatment (13, 14), which may be reflected by changes in circulating neutrophil, lymphocyte, and platelet counts. The D-value used in this study quantified the magnitude of change between baseline and the post-chemotherapy measurement, irrespective of direction. Although larger D-values were associated with poorer OS in the primary analysis, pronounced hematological changes may accompany alterations in the host immune-inflammatory response during chemotherapy. The present observational data, however, do not establish that these changes represent systemic instability or disruption of immune-inflammatory homeostasis, nor do they establish a causal mechanism linking hematological changes to survival.

Our study showed that a higher PLR D-value was associated with poorer OS in the primary categorical analysis, although this association was attenuated after accounting for baseline PLR and clinical factors. As a composite marker, PLR reflects the relative balance between platelet and lymphocyte counts. Platelets play multiple roles in cancer progression and tumor–immune interactions. They can protect circulating tumor cells from natural killer (NK) cell-mediated clearance and hemodynamic shear stress and can release mediators involved in tumor growth, angiogenesis, and epithelial–mesenchymal transition (5, 6). Platelets may also modulate T-cell and NK-cell activity and thereby contribute to tumor immune evasion. These biological effects provide a plausible context for the observed association between PLR changes and outcome; however, platelet activation and function were not directly measured in this study. Therefore, a high PLR D-value should not be interpreted as direct evidence of enhanced platelet-mediated tumor protection, metastatic potential, or immune suppression.

Lymphocytes, including CD8+ cytotoxic T cells, CD4+ helper T cells, and NK cells, are important components of antitumor immunity (20). Their abundance and functional status have been associated with antitumor immune activity and clinical outcomes (7, 8). Changes in lymphocyte populations during treatment may therefore contribute to changes in PLR. Moreover, alterations in lymphocyte subsets, including regulatory T cells, may influence the immunological state of the tumor microenvironment (21). Taken together, PLR provides an indirect measure integrating platelet- and lymphocyte-related components of the systemic immune-inflammatory response. A higher PLR D-value may therefore reflect a greater change in this relative balance during chemotherapy, but the biological basis of the observed association cannot be determined from the present data.

Direction-sensitive analysis provided additional context for interpreting the absolute D-value. Greater positive signed PLR changes were associated with poorer OS in the initial model, although this association was attenuated and became borderline after stratification by chemotherapy regimen. In addition, the simple comparison between patients with increased and decreased PLR was not statistically significant. These findings suggest a possible direction-dependent association, although it was modest and sensitive to model specification.

In addition to PLR D-value, sex, anemia status, and the extent of tumor involvement were associated with survival in patients with NSCLC. Female patients exhibited better outcomes than males, consistent with previously reported sex-related differences in NSCLC survival (22). Patients with anemia had an increased risk of mortality, consistent with previous studies linking anemia with poorer outcomes in NSCLC and other cancer populations (23, 24). Furthermore, patients with whole-lung tumor involvement had poorer survival than those with single-lobe involvement, consistent with the adverse prognostic implications of more extensive intrapulmonary disease.

A prognostic nomogram was constructed to explore whether PLR D-value could be combined with clinical factors for risk estimation. The apparent C-index was 0.630, and the optimism-corrected C-index after 1,000 bootstrap resamples was 0.618, indicating limited discriminative performance. The 5-year AUC was 0.783. Accordingly, the nomogram should be regarded as an exploratory risk-estimation model rather than a tool ready for routine clinical use. External validation in independent cohorts is required before its clinical utility can be established.

Several limitations should be considered. First, the retrospective design may introduce selection bias, and residual confounding cannot be fully excluded. Several clinicopathological variables, particularly clinical stage and tumor size, had substantial missing data. Consequently, analysis requiring complete covariate information were restricted to 214 patients, which may have reduced statistical power and introduced complete-case selection bias. Because OS was measured from the first chemotherapy session, whereas the post-chemotherapy blood measurement was obtained within the subsequent 7 days, a limited guarantee-time interval remained, and residual guarantee-time bias cannot be completely excluded. Second, only two hematological measurements were available, limiting assessment of longitudinal PLR trajectories across multiple chemotherapy cycles. Although direction-sensitive analysis were additionally performed, the association between signed PLR change and OS was weak and sensitive to model specification, whereas the simple increase-versus-decrease comparison was not statistically significant. Third, the optimal cutoff values were derived and evaluated in the same cohort using X-tile, which may introduce data-driven overfitting; therefore, these thresholds require external validation. Importantly, although PLR D-value was associated with OS in the primary categorical analysis, its association was attenuated after adjustment for baseline PLR and clinical factors, indicating that its incremental prognostic value beyond baseline PLR may be limited. Fourth, subsequent anticancer treatments during follow-up were not consistently available for systematic adjustment, and residual treatment-related confounding of OS cannot be excluded. The lack of data on PD-L1 expression and other immunotherapy-related biomarkers further limits the applicability of these findings in the context of contemporary treatment. Treatment intent (adjuvant versus palliative) was not consistently documented and could not be reliably incorporated into stratified analysis. Although no significant interaction between disease stage and PLR D-value was detected, the relatively small number of events in the early-stage subgroup limited the power to detect stage-related heterogeneity. Finally, the nomogram underwent internal validation only, and the optimism-corrected C-index of 0.618 indicates limited discrimination. Larger prospective cohorts with independent external validation are needed to determine the reproducibility and clinical relevance of these findings.

## Conclusion

This study shows that early changes in peripheral blood PLR following the first chemotherapy session are associated with survival outcomes in patients with NSCLC. In the primary categorical analysis, a high PLR D-value was associated with poorer OS; however, this association was attenuated after accounting for baseline PLR and clinical factors, suggesting that its incremental prognostic value may be limited. PLR changes may accompany alterations in systemic immune-inflammatory status, although their biological significance remains to be clarified. The nomogram should be regarded as an exploratory risk-estimation model and requires external validation before clinical application.

## Data Availability

The data that support the findings of this study are available from the corresponding authors upon reasonable request.

## Supplementary material

Supplementary Table 1 Nested Cox regression analysis evaluating the incremental prognostic information of baseline PLR and continuous PLR D-value for overall survival.

Supplementary Table 2 Sensitivity analysis evaluating the prognostic information of categorical PLR D-value after adjustment for baseline PLR and clinical covariates.
