# Supplementary material for: Association of dynamic changes in platelet-to-lymphocyte ratio during chemotherapy with survival in patients with non-small cell lung cancer
Source: Front Oncol. 2026 Oct 2;16:1934583. doi: 10.3389/fonc.2026.1934583 (PMC13672012; doi:10.3389/fonc.2026.1934583)
Supplement: Supplementary file 1 [file Table1.docx]

**Supplementary Table S1. Nested Cox regression analyses evaluating the incremental prognostic information of baseline PLR and continuous PLR D-value for overall survival.**

| Variables | Model 1:  Clinical model HR (95% CI) | *P* | Model 2:  Clinical model + baseline PLR HR (95% CI) | *P* | Model 3:  Clinical model + baseline PLR + PLR D-value HR (95% CI) | *P* |
| --- | --- | --- | --- | --- | --- | --- |
| Sex | | | | | | |
| Male | 1 (ref) | - | 1 (ref) | - | 1 (ref) | - |
| Female | 0.533 (0.328–0.864) | 0.011 | 0.520 (0.321–0.842) | 0.008 | 0.521 (0.322–0.844) | 0.008 |
| Clinical stage | | | | | | |
| Ⅰ | 1 (ref) | - | 1 (ref) | - | 1 (ref) | - |
| Ⅱ | 1.353 (0.285–6.426) | 0.703 | 1.468 (0.309–6.990) | 0.629 | 1.470 (0.309–6.998) | 0.629 |
| Ⅲ | 3.443 (0.825–14.373) | 0.090 | 3.444 (0.825–14.383) | 0.090 | 3.422 (0.819–14.305) | 0.092 |
| Ⅳ | 4.297 (1.044–17.680) | 0.043 | 4.517 (1.098–18.591) | 0.037 | 4.512 (1.096–18.569) | 0.037 |
| Tumor location | | | | | | |
| Single lobe | 1 (ref) | - | 1 (ref) | - | 1 (ref) | - |
| Bilobar involvement | 1.028 (0.703–1.504) | 0.886 | 1.065 (0.727–1.560) | 0.746 | 1.068 (0.729–1.565) | 0.734 |
| Whole lung | 2.256 (1.113–4.570) | 0.024 | 2.384 (1.177–4.830) | 0.016 | 2.379 (1.174–4.821) | 0.016 |
| Maximum tumor diameter | | | | | | |
| <4.16 | 1 (ref) | - | 1 (ref) | - | 1 (ref) | - |
| 4.16-5.20 | 1.353 (0.819–2.234) | 0.238 | 1.467 (0.885–2.432) | 0.138 | 1.461 (0.880–2.424) | 0.142 |
| ≥5.20 | 1.480 (1.009–2.171) | 0.045 | 1.447 (0.986–2.122) | 0.059 | 1.454 (0.990–2.136) | 0.056 |
| Histological type | | | | | | |
| Adenocarcinoma | 1 (ref) | - | 1 (ref) | - | 1 (ref) | - |
| Squamous cell carcinoma | 0.804 (0.550–1.177) | 0.263 | 0.774 (0.528–1.135) | 0.190 | 0.779 (0.530–1.145) | 0.203 |
| Anemia status | | | | | | |
| No | 1 (ref) | - | 1 (ref) | - | 1 (ref) | - |
| Yes | 1.739 (1.180–2.564) | 0.005 | 1.543 (1.027–2.317) | 0.037 | 1.547 (1.029–2.324) | 0.036 |
| Chemotherapy regimen | | | | | | |
| Monotherapy | 1 (ref) | - | 1 (ref) | - | 1 (ref) | - |
| Combination therapy | 0.773 (0.529–1.129) | 0.183 | 0.816 (0.556–1.198) | 0.300 | 0.810 (0.551–1.192) | 0.285 |
| Baseline PLR, per 50-unit increase | - | - | 1.084 (1.010–1.164) | 0.026 | 1.078 (0.994–1.168) | 0.069 |
| PLR D-value, per 50-unit increase | - | - | - | - | 1.010 (0.947–1.078) | 0.762 |
| Model characteristics | | | | | | |
| AIC | 1423.667 |  | 1421.073 |  | 1422.983 |  |
| C-index | 0.680 |  | 0.679 |  | 0.681 |  |
| Likelihood-ratio test vs preceding model | - | - | *χ²* = 4.594 | 0.032 | *χ²* = 0.090 | 0.764 |

**Notes:** All three nested Cox regression models were fitted in the same complete-case population of 214 patients, including 156 deaths. Model 1 included sex, clinical stage, tumor location, maximum tumor diameter, histological type, anemia status, and chemotherapy regimen. Model 2 additionally included baseline PLR, and Model 3 additionally included continuous PLR D-value. Baseline PLR and PLR D-value were modeled per 50-unit increase. Incremental model fit was evaluated using likelihood-ratio tests, with Model 2 compared with Model 1 and Model 3 compared with Model 2. Patients with missing values for any covariate required for these models were excluded from the complete-case analysis. HR, hazard ratio; CI, confidence interval; PLR, platelet-to-lymphocyte ratio; AIC, Akaike information criterion; C-index, concordance index; ref, reference category.

**Supplementary Table S2. Sensitivity analysis evaluating the prognostic information of categorical PLR D-value after adjustment for baseline PLR and clinical covariates**

| Variables /  model characteristics | Model 2:  Clinical model + baseline PLR | Model 3:  Clinical model + baseline PLR + categorical PLR D-value |
| --- | --- | --- |
| Baseline PLR, per 50-unit increase | | |
| HR (95% CI) | 1.084 (1.010–1.164) | 1.06 (0.987–1.15) |
| *P* | 0.026 | 0.104 |
| PLR D-value | | |
| Low (<45.2) | - | 1 (ref) |
| High (≥45.2) | - | 1.31 (0.911–1.89) |
| P | - | 0.145 |
| Model characteristics | | |
| AIC | 1421.073 | 1420.915 |
| C-index | 0.679 | 0.686 |
| Likelihood-ratio test vs Model 2 | - | *χ²* = 2.159, *P* = 0.142 |

**Notes:** This sensitivity analysis used the original X-tile-derived PLR D-value cutoff of 45.2. Both models were fitted in the same complete-case population of 214 patients, including 156 deaths, and were adjusted for the same clinical covariates: sex, clinical stage, tumor location, maximum tumor diameter, histological type, anemia status, and chemotherapy regimen. Model 2 included baseline PLR, whereas Model 3 additionally included categorical PLR D-value. Baseline PLR was modeled per 50-unit increase. In the complete-case population, 78 patients were classified as having a Low PLR D-value (<45.2), including 53 deaths, and 136 were classified as having a High PLR D-value (≥45.2), including 103 deaths. Incremental model fit after addition of categorical PLR D-value was evaluated using the likelihood-ratio test. HR, hazard ratio; CI, confidence interval; PLR, platelet-to-lymphocyte ratio; AIC, Akaike information criterion; C-index, concordance index; ref, reference category.
